# Atopy and autoimmune thyroid diseases: melatonin can be useful?

**DOI:** 10.1186/s13052-016-0305-0

**Published:** 2016-11-04

**Authors:** Gabriella D’Angelo, Lucia Marseglia, Sara Manti, Laura Colavita, Caterina Cuppari, Pietro Impellizzeri, Salvatore Arena, Teresa Arrigo, Carmelo Salpietro, Eloisa Gitto

**Affiliations:** 1 Neonatal Intensive Care Unit, Department of Pediatrics, University of Messina, Via Consolare Valeria 1, 98125 Messina, Italy; 2 Neonatal Intensive Care Unit, Department of Pediatrics, University of Messina, Via Consolare Valeria 1, 98125 Messina, Italy; 3 Unit of Paediatric Surgery, Department of Paediatrics, University of Messina, Via Consolare Valeria 1, 98125 Messina, Italy

**Keywords:** Thyroid autoimmunity, Allergic diseases, Dysregulated immune system, Melatonin

## Abstract

Recently, there has been growing interest in the relationship between allergic and autoimmune diseases. Allergy and autoimmunity can be considered two potential outcomes of dysregulated immunity and analysis of literature data shows a strong positive association between a history of Th2-mediated allergic disorders and Th1-mediated autoimmune disorders.

Autoimmune thyroid diseases are the most common of all autoimmune pathological conditions.

Currently, the mechanisms explaining an association among atopy, autoimmunity, and thyroid diseases are not fully understood.

There are data in literature pointing to the relationship between melatonin and thyroid activity. Several studies have suggested a paracrine role for this molecule in the regulation of thyroid activity, documenting that administration, as an antioxidant, in thyroid tissues under conditions of increased oxidative stress, could be helpful to reduce the oxidative processes involved in autoimmune thyroid diseases.

Although thyroid autoimmunity has been regularly associated with atopic conditions in children, the possible protective role of melatonin has not yet been investigated.

This review summarizes what is known regarding the connection between atopy and autoimmune thyroid diseases, and analyses the probable beneficial action of melatonin.

## Background

Hypersensitivity reactions of the immune system have been categorized into atopic and autoimmune reactions, depending on whether the antigen triggering the reaction is endogenous or exogenous, types of cellular and humoral components involved, and clinical symptoms [1].

Atopy is an individual or familial tendency to produce IgE antibodies in response to low doses of allergens and to develop typical symptoms such as atopic dermatitis, allergic rhinitis, and asthma [2, 3]. Atopic diseases have increased in frequency in recent decades and now affect approximately 20 % of the population worldwide [4]. The risk of developing atopic diseases is complex and is strongly influenced by both genetic and environmental factors [5, 6].

Atopy and autoimmunity are two potential outcomes of dysregulated immunity [7].

The incidence of autoimmune diseases has increased in recent decades. Although these conditions have a multifactorial pathogenesis, one generally accepted model is that immune dysregulation, secondary to an infectious process or excessive exposure to an inciting or cross-reactive antigen, promotes a T helper (Th) 1 response leading to progressive inflammation and autoimmunity. Reciprocal counter-regulation of Th1 and Th2 cells predicts that Th1-type autoimmune disease and Th2-mediated allergic disease would occur in mutually exclusive populations of patients [8].

However, data literature are controversial regarding true associations between atopy and autoimmune disease [9–12]. Recent observations have identified additional lymphocyte subsets, such as Th17 cells [9], soluble factors such as IL-9 [10] and regulatory T cells (T reg) [11] as a common link between atopy and autoimmunity. It has been reported that infantile atopy increases a predisposition to autoimmune disorders, suggesting that these two entities might have immune pathways, common to both pathological conditions, justifying partially the increased prevalence and/or the co-presence of atopic and autoimmune diseases [13, 14].

Conversely, Skaaby et al. [12] did not find statistically significant associations between atopic predisposition and autoimmune disorders, although they could not exclude moderate effects of atopy on autoimmune disease.

The thyroid gland is the most common organ affected by autoimmune disease. Autoimmune phenomena, especially thyroid autoimmunity, have often been associated with other autoimmune diseases, such as chronic urticaria in adults [15] and children [16].

Although thyroid autoimmunity has regularly been associated with atopy in children, the possible protective role of antioxidants, such as melatonin, has not yet been fully investigated, although its role has been documented in other atopic conditions, such as dermatitis and asthma [17, 18].

Melatonin plays key roles in several important physiological functions, including neuroimmunological actions and immunomodulatory effects in allergic diseases, although its potential use in atopic patients has rarely been considered [19].

Experimental data [17] suggested that melatonin inhibits development of atopic eczema, a condition accompanied by infiltration and activation of mast cells with release vasoactive and proinflammatory mediators, and reduces serum total IgE and IL-4.

Furthermore, emerging evidence suggest that melatonin represents a potent antioxidant and protective substance in skin photobiology [20, 21]. It has been reported that melatonin also protects keratinocytes against ultraviolet (UV) radiation B-induced oxidative stress and DNA damage and that endogenous intracutaneous melatonin production, together with topically-applied exogenous melatonin, would represent one of the most potent anti-oxidative defense systems against the UV-induced damage to the skin [22].

Melatonin, therefore, has been hypothesized as a potential therapeutic option also in atopic dermatitis (AD), protecting skin integrity and helping to maintain a functional epidermal barrier [23, 24].

Moreover, it has been documented that melatonin, regulating smooth muscle tone and influencing the immune response in allergic asthma, could be implicated in regulating bronchial hyper-responsiveness [25, 26]. Furthermore, melatonin may, however, act as a pro-inflammatory agent in asthma leading to bronchial constriction [17, 25].

There are some data in literature pointing to the relationship between melatonin and thyroid activity and its involvement in the hypothalamic-pituitary-thyroid axis [27].

This review summarizes what is known regarding the connection between atopy and autoimmune thyroid diseases and analyses the possible protective role of melatonin.

### A link between atopy and autoimmune thyroid disease

It has been proposed that innate immunity has a role in allergic inflammation, and epithelial cells, dendritic cells, natural killer lymphocytes, and mast cells might play a regulatory role in allergy. It has also been suggested that there is some form of cooperation between Th2 mediated allergy, and Th1 mediated inflammation responses [28].

The adaptive cellular immune response has been characterized broadly as being polarized in two directions [29]. Type 1 responses, directed by Th1 CD4+ T cells and identified by the signature cytokine interferon (IFN)-g, are considered as protective against infections by intracellular pathogens [30], and have been incriminated in the pathogenesis of autoimmune diseases, such as thyroid disease. Many other autoimmune diseases such as rheumatoid arthritis, juvenile rheumatoid arthritis, insulin-dependent diabetes mellitus and multiple sclerosis are associated with a Th1 phenotype [31].

Autoimmune thyroid disease (ATD) is a multifactorial disease in which autoimmunity against thyroid antigens develops against a particular genetic background facilitated by exposure to environmental factors. Autoimmune thyroid diseases are characterized by infiltration of the thyroid by T and B cells which are reactive to thyroid antigens, by the production of thyroid autoantibodies and by abnormal thyroid function [32]. ATD presents a hereditary component, as reported in several studies that show a definite genetic propensity for thyroid autoimmunity to run in families [33]. The childhood prevalence of chronic autoimmune thyroiditis peaks in early to mid-puberty [34].

**Fig. 1:**
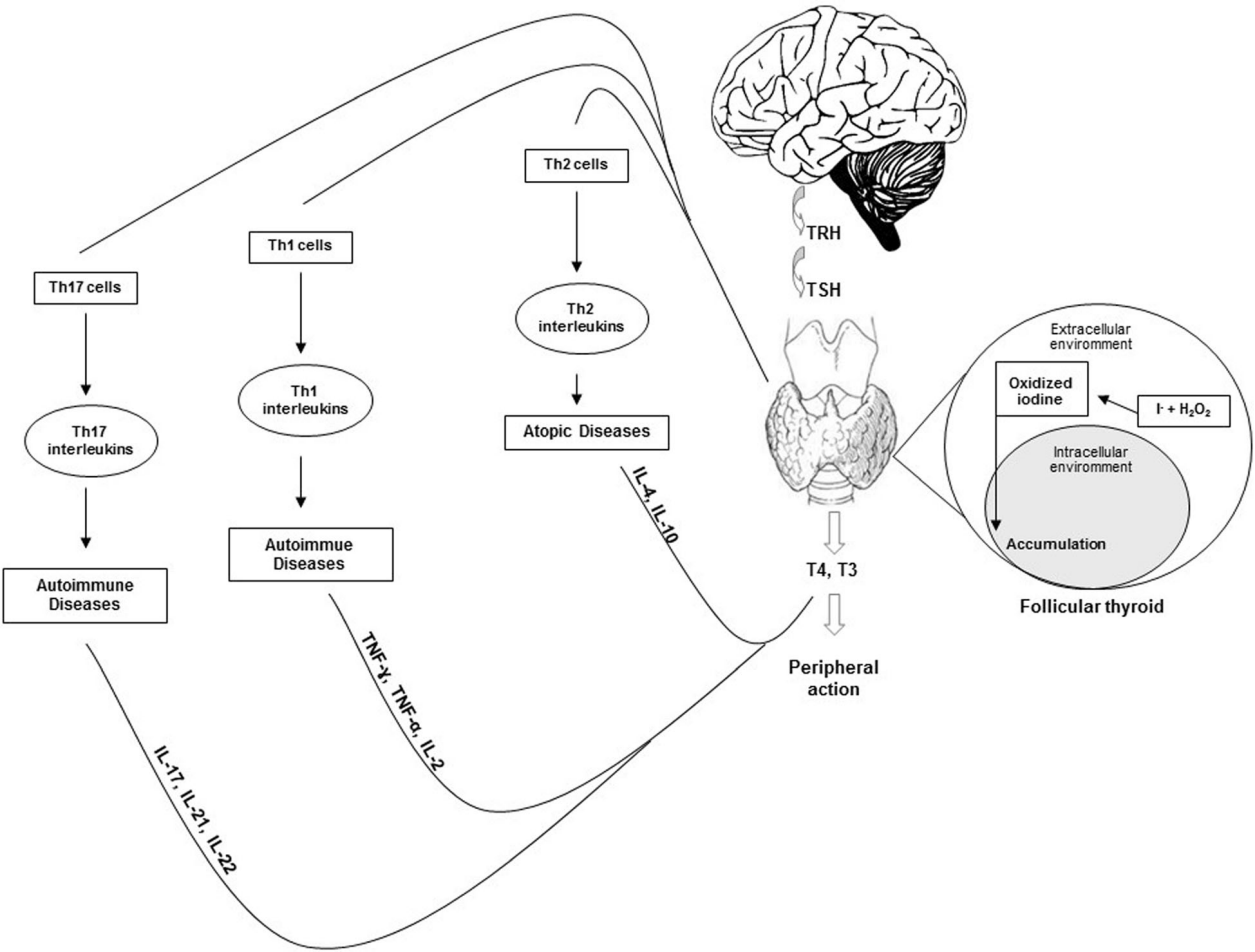
Th1 - Th2 - Th17 response and respective cytotoxic and humoral involvement in inflammation and autoimmunity

By contrast, type 2 responses, directed by Th2 CD4+ T cells and identified by the signature cytokines interleukin (IL) -4, IL-5 and IL-13, are considered to play a pathogenic role in allergic diseases [35]. Reciprocal counter-regulation of Th1 and Th2 cells [36] predicts that Th1-type autoimmune diseases and Th2-mediated allergic diseases would occur in mutually exclusive populations of patients. However, recent observations have challenged the validity of the long-standing Th1/Th2 paradigm [37], and a far more complex story explaining the immunological basis of cellular immune-mediated host defence and the pathogenesis of autoimmune and allergic diseases is emerging. The new paradigm identifies additional lymphocyte subsets, such as Th17 T cells, Treg and novel soluble factors. These provide a new prism through which to examine the intersection of autoimmune and allergic disease (Fig. 1) [37].

Mittermann et al. demonstrated that a considerable percentage of patients suffering from atopic dermatitis had enhanced IgE autoantibody levels against a broad variety of human proteins expressed in a variety of cell and tissue types. Furthermore, they showed that the levels of IgE autoantibodies were associated with severity of disease [38].

Several studies reported a higher incidence of ATD in subjects affected by allergic diseases. Pedullá et al. showed an increased prevalence of thyroid autoimmunity in children with AD, underlining a significant relationship between the expression of AD and thyroid autoimmunity in a pediatric population [39]. Furthermore, Pedullá et al. demonstrated that the frequency of thyroid autoimmunity was significantly higher among children with IgE-mediated AD than non-IgE-mediated AD, suggesting atopy and thyroid autoimmunity as potential outcomes of dysregulated immunity [39]. Hidaka et al. suggested that eosinophils and their activation play an important role in inflammatory processes in allergic diseases. Th2 cytokines stimulate eosinophils and it has recently been found that the peripheral eosinophil count was increased in thyrotoxic patients with Graves’ disease [40]. Moreover, the same authors demonstrated that an allergic condition is closely related to Graves’ disease and that a Th2-type immune response is crucial in the pathogenesis of Graves’ disease [41].

### Use of antioxidants in thyroid diseases: melatonin

Whereas oxidative reactions occur in all tissues and organs, the thyroid gland is an organ in which oxidative processes are widely signalized and are indispensable for physiological functions of the organism [42]. Hydrogen peroxide acts as an electron acceptor at each step of thyroid hormone biosynthesis and is essential for thyroperoxidase (TPO) activity, the key enzyme in this process. In addition, antioxidative enzyme activity [superoxide dismutase (SOD), glutathione (GSH) peroxidase (GSH-Px) and catalase (CAT)] has been well documented in the thyroid [43].

The use of various exogenous antioxidants in thyroid tissues has been shown to be beneficial in experimental (in vitro or in vivo) studies [43, 44]. Unfortunately, the applicability of different antioxidants has not yet been clearly confirmed in clinical studies and still no effective antioxidative medication is available for widespread use [45].

Moreover, melatonin, an ubiquitous antioxidant molecule with pleiotropic action in many tissues, has protective effects against oxidative stress [46, 47], the thyroid included [48].

There are data in literature pointing to the relationship between melatonin and thyroid activity. Several studies have suggested a paracrine role for this molecule in the regulation of thyroid activity. The antioxidant role of melatonin in the thyroid gland was first reported by Kvetnoy [49]. It has been demonstrated that melatonin is, at rat thyroid level, synthesized by C cells -the minor neuroendocrine thyroid cell population- and, that melatonin receptors (MT)1 are present in follicular cells [50], suggesting a role for thyroid melatonin. Recently, another study has shown that thyroid C-cells synthesize melatonin under thyroid-stimulating hormone control, supporting the hypothesis of a paracrine role for C-cell-synthesised melatonin within the thyroid gland.

In particular, Garcia-Marin et al. [27] analyzed both the effect of melatonin on the expression of the thyroid tissue-specific genes PAX8 and TTF1 and examined TSH regulation of key enzymes for melatonin biosynthesis, such as aralkylamine N-acetyltransferase (AANAT) and acetyl serotonin methyltransferase (ASMT). They showed both the involvement of melatonin in thyroid function by directly-regulating thyroglobulin gene expression in follicular cells and that AANAT and ASMT are regulated by thyroid-stimulating hormone. Furthermore, it has been demonstrated that thyroid C-cells synthesized melatonin under thyroid-stimulating hormone control, supporting the possible paracrine role for C-cell-synthesised melatonin within the thyroid [27].

Additionally, Karbownik M. et al. commented the evidence that melatonin, acting as an antioxidant agent, influences thyroid growth, counteracting significant oxidative damage that occurs in the course of thyroid diseases, and the stimulatory effects of thyroid hormones [42].

The same research group [48], in an experimental study, estimated melatonin effects on basal and iron-induced lipid peroxidation related to Fenton reaction in homogenates of the porcine thyroid gland, showing that the degree of lipid peroxidation was decreased, in a concentration-dependent manner, by melatonin, and supporting the protective role of this indolamine in preventing pathological processes, such as thyroid cancer, related to oxidative injury.

Data in literature has shown melatonin action on the thyroid gland itself, such as the inhibitory effect of melatonin on cell proliferation and thyroid hormone synthesis [51, 52] or the protective effect of melatonin against oxidative damage in the thyroid gland [53, 54].

It has been well reported that the administration of chronic melatonin is efficient in protecting against the damaging effects of different prooxidants and that under basal conditions it does not change the level of oxidative damage to macromolecules [55]. In addition, it has been evaluated that the effects of Caffeic acid phenethyl ester (CAPE) on Fenton reaction-induced oxidative damage to membrane lipids (lipid peroxidation, LPO) in porcine thyroid and the liver are similar to those caused by melatonin. Whereas CAPE decreased basal LPO in a concentration-dependent manner in both tissues, melatonin did not change the basal LPO level. When antioxidants were used together with Fenton reaction substrates, they prevented – in a concentration-dependent manner and to a similar extent – experimentally- induced LPO in both tissue effects of melatonin [56].

## Conclusions

Regulatory T cells are important components of the homeostasis of the immune system, as impaired regulatory T cell activity can cause autoimmune diseases and atopy [57].

Atopy and autoimmunity are manifestations of immune system dysfunction and are referred to as distinct immunological reactions with common pathogenic mechanisms [58].

The mechanism whereby ATD is associated with allergic diseases is poorly understood. Several researchers have observed serum histamine releasing activity in a subgroup of adult patients with chronic urticaria, which was attributable to an IgG autoantibody directed against the alpha chain of the high affinity IgE receptor of the mast cells or, less commonly, against IgE itself [59, 60].

Rottem M. et al. [61] affirmed that atopic skin manifestations and thyroid autoimmunity “most likely are associated, parallel autoimmune events,” whereas Dreskin SC. et al. concluded that [62] “there are no compelling arguments to decide whether or not thyroid autoimmunity plays a significant role in the pathogenesis of chronic urticaria.”

It is important to identify infants at risk for developing lifelong chronic atopic and autoimmune diseases and utilize the critical window of opportunity early in life for therapeutic intervention that targets Th1- or Th2-specific responses revealing atopy or autoimmunity respectively.

During the last decade, wide evidence has contributed to confirm the antioxidant and protective role played by melatonin in the normal, as well as in the prooxidant-agent insulted, thyroid-gland [43, 48, 63].

Finally, it has been documented that the administration of melatonin, as an antioxidant, in thyroid tissue under condition of increased oxidative stress, could be helpful to reduce oxidative processes involved in autoimmune thyroid diseases [27, 42, 48].

Melatonin is a molecule with an excellent biosafety profile, widely used for decades in some countries, and no serious side effects or treatment-related complications with short or long-term melatonin therapy in children and adults have been reported [64].

In addition, further studies are necessary to elucidate the underlying mechanisms and the common pathways involved in atopy and thyroid autoimmune diseases.

Therefore, melatonin could be hypothesized as a potentially useful agent in the treatment of ATD, but further studies are necessary to clarify the immunological-hormonal effects of this indolamine.

## Abbreviations

AANAT: Aralkylamine N-acetyltransferase
AD: Atopic dermatitis
ASMT: Acetyl serotonin methyltransferase
ATD: Autoimmune thyroid disease
CAPE: Caffeic acid phenethyl ester
CAT: Catalase
GSH: Glutathione
GSH-Px: Glutathione peroxidase
IFN-g: Interferon
IL: Interleukin
LPO: Lipid peroxidation
MT: Melatonin receptors
ROS: Reactive oxygen species
SOD: Superoxide dismutase
T reg: Regulatory T cells
Th: T helper
TPO: Thyroperoxidase
